# Supplementary material for: Floquet optical selection rules in black phosphorus
Source: Sci Adv. 2025 Aug 20;11(34):eadw2744. doi: 10.1126/sciadv.adw2744 (PMC12366682; doi:10.1126/sciadv.adw2744)
Supplement: Supplementary file 1 — Notes S1 to S4 Tables S1 to S5 Figs. S1 to S8 References [file sciadv.adw2744_sm.pdf]

Science Advances
AAAS

Supplementary Materials for

Floquet optical selection rules in black phosphorus

Benshu Fan *et al.*

Corresponding author: Wenhui Duan, duanw@mail.tsinghua.edu.cn; Angel Rubio, angel.rubio@mpsd.mpg.de; Peizhe Tang, peizhet@buaa.edu.cn

Sci. Adv. **11**, eadw2744 (2025)
DOI: 10.1126/sciadv.adw2744

This PDF file includes:

Notes S1 to S4
Tables S1 to S5
Figs. S1 to S8
References

1 Supplementary Note 1: Symmetry analysis of black phosphorus

The monolayer black phosphorus (BP) and BP thin film have the same in-plane translation symmetry, and the point group of the space group is D_{2h} for both of them, as shown in Table S1.

Table S1: The group of the BP thin film and monolayer BP.

	Space group	Point group
BP thin film	64 ($Cmce$)	8 (D_{2h})
monolayer BP	53 ($Pmna$)	8 (D_{2h})

Following the convention of Refs. (*57,62*), we choose the armchair (AC) direction as x direction and the zigzag (ZZ) direction as y direction of the monolayer BP. As shown in Fig. S1, there is only one glide-mirror plane (green plane) in the monolayer BP (*43*), and under the glide-mirror operation, the monolayer BP is invariant.

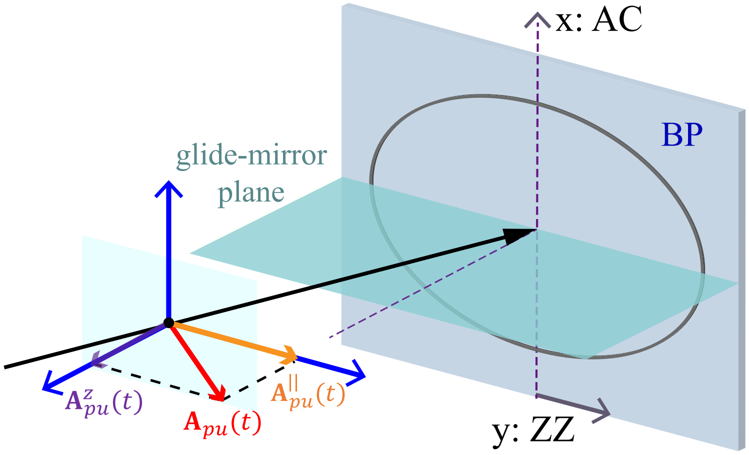

Figure S1: Schematic of the monolayer BP under the laser pumping. The vector potential of the pumping laser is $\mathbf{A}_{pu}(t)$, with its in-plane (out-of-plane) component $\mathbf{A}^{\|}_{pu}(t)$ ($\mathbf{A}^{z}_{pu}(t)$). The green plane perpendicular to the x-axis is the glide-mirror plane.

At the Γ point, the valence band (VB) and conduction band (CB) wavefunctions, denoted as $|\psi^v\rangle$ and $|\psi^c\rangle$, are dominated by p_z orbitals. These wavefunctions transform as the irreducible

representations Γ_2^+ and Γ_4^-, respectively (*57*). Since the wave vector group at the Γ point is just the Abelian D_{2h} group, according to the character table of the point group D_{2h} in Table S2, the characters of the irreducible representations Γ_2^+ and Γ_4^- respect to the mirror operation M_{yz} are -1 and 1, indicating $|\psi^v\rangle$ ($|\psi^c\rangle$) is **odd** (**even**) with respect to the glide-mirror plane.

Actually, the pseudospin degree of freedom in monolayer BP arises from the superposition of wavefunctions $|A\rangle$ and $|B\rangle$ at A and B sublattices, and at the Γ point the VB and CB wavefunctions $|\psi^v\rangle \simeq |A\rangle - |B\rangle$ and $|\psi^c\rangle \simeq |A\rangle + |B\rangle$ exhibit opposite pseudospin.

Table S2: The character table for the point group D_{2h}.

	E	C_{2x}	C_{2y}	C_{2z}	I	M_{yz}	M_{xz}	M_{xy}	Basis
Γ_1^+	1	1	1	1	1	1	1	1	x^2, y^2, z^2
Γ_2^+	1	-1	1	-1	1	-1	1	-1	R_y, xz
Γ_3^+	1	1	-1	-1	1	1	-1	-1	R_x, yz
Γ_4^+	1	-1	-1	1	1	-1	-1	1	R_z, xy
Γ_1^-	1	1	1	1	-1	-1	-1	-1	xyz
Γ_2^-	1	-1	1	-1	-1	1	-1	1	y
Γ_3^-	1	1	-1	-1	-1	-1	1	1	x
Γ_4^-	1	-1	-1	1	-1	1	1	-1	z

2 Supplementary Note 2: An investigation of the photoemission spectroscopy via group theory

2.1 Preliminary

The matrix element effect is based on Fermi's golden rule in quantum mechanics, which has a strong relation with initial, final states and the light polarization direction. In order to analyze features in the photoemission spectra, the key is to comprehend the matrix element. In this chapter we will use group theory to investigate the matrix element effect of the monolayer BP thoroughly.

Under the velocity gauge, the light-matter interaction Hamiltonian can be written as $\hat{H}' \simeq \mathbf{A}_{pr} \cdot \hat{\mathbf{p}}$, where $\mathbf{A}_{pr}$ is the vector potential of the external probe laser and $\hat{\mathbf{p}}$ is the momentum operator of the electron in the monolayer BP (*63*). So the matrix element can be defined as

$$\mathcal{M} = \langle \psi_f | \hat{H}' | \psi_i \rangle \simeq \mathbf{A}_{pr} \cdot \langle \psi_f | \hat{\mathbf{p}} | \psi_i \rangle \tag{S1}$$

where $|\psi_i\rangle$ ($\left|\psi_f\right\rangle$) is the initial (final) state wavefunction. Once the analyser slit is positioned orthogonal to the glide-mirror plane (yz plane) of the monolayer BP, the final state wavefunction $\left|\psi_f\right\rangle$ must be **even** for this plane (*58*).

If we want the non-zero photoemission intensity, the matrix element $\mathcal{M}$ must be even function under the mirror operation M_{yz}, and because the final state wavefunction $\left|\psi_f\right\rangle$ is **even**, the direct product between the group representations of $\hat{\mathbf{p}}$ and $|\psi_i\rangle$ should be **even** under the mirror operation M_{yz}, such as Γ_1^+, Γ_3^+, Γ_2^- and Γ_4^- in Table S2.

For the convenience of the following discussion, we list two symbolic conventions for instance:

- AC (s-$pol.$): The polarization direction of the probe laser is totally along the AC (x) direction, namely there is no out-of-plane (z) component.

- AC (s-$pol.$)@ZZ (p-$pol.$): The polarization direction of the pumping laser is totally along the AC direction, and the polarization direction of the probe laser is in the glide-mirror plane, namely, there are ZZ (y) and z components.

Next, we delve into a detailed discussion of the matrix elements of angle-resolved photoemission spectroscopy (ARPES) and time- and angle-resolved photoemission spectroscopy (TrARPES).

2.2 The matrix element of ARPES

For the ARPES experiment, we can comprehend the process as: $|\psi_i\rangle \xrightarrow{\mathbf{A}_{pr}} |\psi_f\rangle$. Next we take two cases as examples to analyze the direct product between the representations of $\hat{H}'$ and $|\psi_i\rangle$.

Case 1 — AC (s-$pol.$):

The vector potential $\mathbf{A}_{pr}$ of the probe laser is along the AC (x) direction, namely, $\hat{H}' \simeq A^x_{pr}\hat{p}_x$ transforms as the irreducible representation Γ_3^- (**odd**):

1. If the initial state $|\psi_i\rangle$ is the VB wavefunction $|\psi^v\rangle$ with the irreducible representation Γ_2^+, then we can obtain the irreducible representation of $\hat{H}'|\psi^v\rangle$ as

$$\underbrace{\Gamma_3^-}_{probe} \otimes \underbrace{\Gamma_2^+}_{|\psi^v\rangle} = \Gamma_4^- \tag{S2}$$

which is **even**. So around the Γ point, the VB can be detected under the AC (s-$pol.$) probe laser. We can also intuitively comprehend why the matrix element $\mathcal{M}$ is nonzero by considering symmetry. Under the M_{yz} symmetry operation, the coordinates transform as $(x, y, z) \xrightarrow{M_{yz}} (-x, y, z)$, and the wavefunctions transform as $M_{yz}|\psi^v\rangle = \chi^v|\psi^v\rangle$ and $M_{yz}|\psi_f\rangle = \chi_f|\psi_f\rangle$, where χ^v and χ_f are the eigenvalues of $|\psi^v\rangle$ and $|\psi_f\rangle$. So we can rewrite the matrix element $\mathcal{M}$ as

$$\begin{aligned}\mathcal{M} &\simeq A^x_{pr}\langle\psi_f|\hat{p}_x|\psi^v\rangle \\ &= A^x_{pr}\left(\langle\psi_f|M^\dagger_{yz}\right)\left(M_{yz}\hat{p}_x M^\dagger_{yz}\right)\left(M_{yz}|\psi^v\rangle\right) \\ &= -(\chi^*_f\chi^v)A^x_{pr}\langle\psi_f|\hat{p}_x|\psi^v\rangle = -(\chi^*_f\chi^v)\mathcal{M}\end{aligned} \tag{S3}$$

It follows that the matrix element $\mathcal{M}$ vanishes if $(\chi^*_f\chi^v) = 1$. Based on our earlier analysis, the final state $|\psi_f\rangle$ is even under M_{yz} operation with $\chi_f = 1$, and the VB wavefunction $|\psi^v\rangle$ at the Γ point is odd with $\chi^v = -1$. Therefore, $(\chi^*_f\chi^v) = -1$ and the matrix element $\mathcal{M}$ is nonzero. As a case study, we calculated the ARPES intensity plot along the AC direction for the monolayer BP under the AC probe laser, as shown in Fig. S2A. The signals of the VB around the Γ point are nonzero, consistent with the theoretical analysis.

2. If the initial state $|\psi_i\rangle$ is the CB wavefunction $|\psi^c\rangle$ with the irreducible representation Γ_4^-, in the same way we obtain the irreducible representation of $\hat{H}'|\psi^c\rangle$ as $\Gamma_3^- \otimes \Gamma_4^- = \Gamma_2^+$, which is **odd**. So around the Γ point, the CB can't be detected under the AC (s-$pol.$) probe laser.

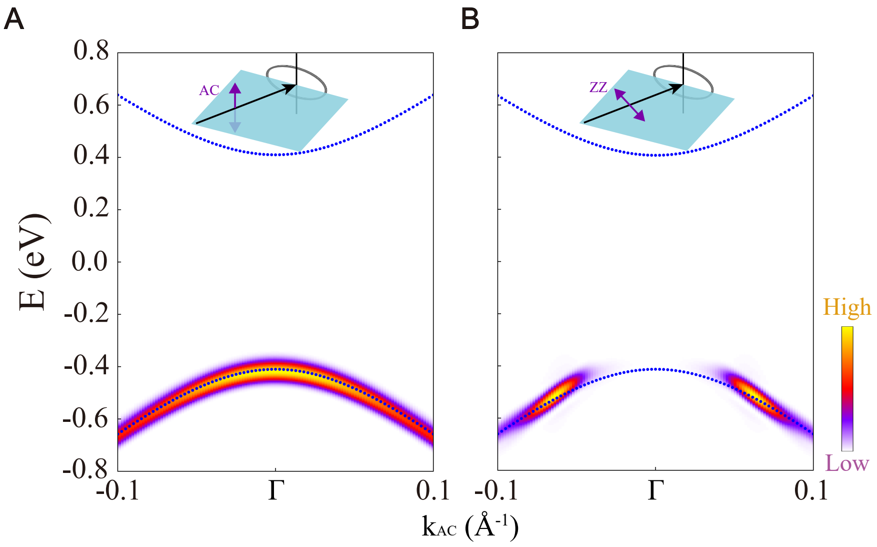

Figure S2: The simulated ARPES intensity plots in equilibrium. (**A**) The ARPES intensity plot calculated along the AC direction for the monolayer BP without any doping. The polarization of the probe laser is along the AC (s-$pol.$) direction as depicted in the inset. During the calculations, the photon energy $\hbar\omega$ of the probe laser is selected to be 6.2 eV, and the peak intensity of the probe laser is 0.03×10^9 W/cm^2 with a pulse duration T_{pr} of 100 fs. (**B**) Similar results as (A) but the polarization of the probe laser is along the ZZ (p-$pol.$) direction. The equilibrium band structures are in blue dot lines for both panels.

Case 2 — ZZ (p-$pol.$):

The vector potential $\mathbf{A}_{pr}$ of the probe laser is in the scattering plane, then the representation of $\hat{H}'$ is $\Gamma_2^- \oplus \Gamma_4^-$ (**even**):

1. If the initial state $|\psi_i\rangle$ is the VB wavefunction $|\psi^v\rangle$ with the irreducible representation Γ_2^+, then $(\Gamma_2^- \oplus \Gamma_4^-) \otimes \Gamma_2^+ = \Gamma_1^- \oplus \Gamma_3^-$, which is **odd**. So around the Γ point, the VB can't be detected under the ZZ (p-$pol.$) probe laser, as shown in Fig. S2B. The parameters are the same as the AC probe laser, but the polarization direction $\boldsymbol{\epsilon}_{pr}$ is $(0, 1, 1)$ for the ZZ probe laser, corresponding to a 45° incident angle. Importantly, the symmetry-governed spectral features are insensitive to the choice of incident angle.

2. If the initial state $|\psi_i\rangle$ is the CB wavefunction $|\psi^c\rangle$ with the irreducible representation Γ_4^-, then $(\Gamma_2^- \oplus \Gamma_4^-) \otimes \Gamma_4^- = \Gamma_3^+ \oplus \Gamma_1^+$, which is **even**. So around the Γ point, the CB can be detected

under the ZZ (p-$pol.$) probe laser.

In summary, we list symmetry analyses of the matrix element $\mathcal{M}$ under different conditions for the probe laser in Table S3. Moreover, we also add some experimental evidence to confirm the rationality of our analyses.

Table S3: The symmetry analysis of the ARPES matrix element.

	Probe laser		Matrix element $\mathcal{M}$				Experimental evidence in references
	Polarization direction	Group representation	$\lvert\psi^v\rangle$ group representation	$\hat{H}'\lvert\psi^v\rangle$ group representation	$\lvert\psi^c\rangle$ group representation	$\hat{H}'\lvert\psi^c\rangle$ group representation	
1	AC (s-$pol.$)	Γ_3^-	Γ_2^+	$\Gamma_3^- \otimes \Gamma_2^+$, even	Γ_4^-	$\Gamma_3^- \otimes \Gamma_4^-$, odd	(*43*), Fig. 3A (*38*), Extended Data Fig. 3I
2	ZZ (p-$pol.$)	$\Gamma_2^- \oplus \Gamma_4^-$	Γ_2^+	$(\Gamma_2^- \oplus \Gamma_4^-) \otimes \Gamma_2^+$, odd	Γ_4^-	$(\Gamma_2^- \oplus \Gamma_4^-) \otimes \Gamma_4^-$, even	(*43*), Fig. 3B

2.3 The matrix element of TrARPES

In order to provide a comprehensive physical understanding in this section, we start our analysis from the VB wavefunction $|\psi^v\rangle$. For the TrARPES experiment, we can comprehend the process as $|\psi^v\rangle \xrightarrow{\mathbf{A}_{pu}} |\psi_n^v\rangle \xrightarrow{\mathbf{A}_{pr}} |\psi_f\rangle$, where $|\psi_n^v\rangle$ is the wavefunction of the n-th sideband for the VB. So the key is to obtain the representation of $|\psi_n^v\rangle$.

Now, we consider the simplest effective Hamiltonian of the VB edge around the Γ point for the monolayer BP as

$$\hat{H}_\Gamma(\mathbf{k}_{||}) = -\sum_{i=x,y} \frac{\hbar^2 k_i^2}{2m_i} \tag{S4}$$

where $\mathbf{k}_{||} = (k_x, k_y)$ is the in-plane momentum and m_i is the effective mass on the VB edge at the Γ point.

Floquet states — For the pumping laser which only has in-plane components, we can disregard the in-plane momenta of photoemission electrons since we are primarily concerned with the physics around the Γ point. Consequently, we can focus solely on the Floquet states. Subject to the light-matter coupling in the Peierls substitution ($\mathbf{k}_{||} \to \mathbf{k}_{||} + \mathbf{A}_{pu}(t)$), we can obtain the time-dependent effective Hamiltonian $\hat{H}_\Gamma(t, \mathbf{k}_{||})$ as

$$\hat{H}_\Gamma(t, \mathbf{k}_{||}) = \hat{H}_\Gamma(\mathbf{k}_{||}) + \mathbf{v}(\mathbf{k}_{||}) \cdot \mathbf{A}_{pu}(t) \tag{S5}$$

where $\mathbf{v}(\mathbf{k}_{\|}) = \nabla_{\mathbf{k}} \hat{H}_{\Gamma}(\mathbf{k}_{\|}) = -\left(\frac{\hbar^2 k_x}{m_x}, \frac{\hbar^2 k_y}{m_y}\right)$ and $\mathbf{A}_{pu}(t)$ is the vector potential of the pumping laser. The high-order term $\mathbf{A}_{pu}^2(t)$ has been dropped from our considerations, as it doesn't couple with $\mathbf{k}_{\|}$ and thus doesn't influence the symmetry analysis outlined below. Now we consider the dynamical evolution of $\left|\Psi^{v}(0, \mathbf{k}_{\|})\right\rangle = |\psi^{v}\rangle$ via Schrödinger equation as

$$i\hbar \frac{\partial \left|\Psi^{v}(t, \mathbf{k}_{\|})\right\rangle}{\partial t} = \hat{H}_{\Gamma}(t, \mathbf{k}_{\|}) \left|\Psi^{v}(t, \mathbf{k}_{\|})\right\rangle \tag{S6}$$

and we can obtain the analytical solution as

$$\left|\Psi^{v}\left(t, \mathbf{k}_{\|}\right)\right\rangle = \exp\left\{-\frac{i}{\hbar}\left[\hat{H}_{\Gamma}(\mathbf{k}_{\|})t + \int \mathbf{v}(\mathbf{k}_{\|}) \cdot \mathbf{A}_{pu}(t)dt\right]\right\} |\psi^{v}\rangle \tag{S7}$$

The vector potential $\mathbf{A}_{pu}(t)$ can be written as $\mathbf{A}_{pu}(t) = \boldsymbol{\epsilon}_{pu} A_{pu} \cos \Omega t = \mathbf{A}_{pu} \cos \Omega t$, then the wavefunction in Eq. S7 can be transformed to

$$\begin{aligned}\left|\Psi^{v}\left(t, \mathbf{k}_{\|}\right)\right\rangle &= \exp\left\{-\frac{i}{\hbar}\left[\hat{H}_{\Gamma}(\mathbf{k}_{\|})t + \frac{\mathbf{v}(\mathbf{k}_{\|}) \cdot \mathbf{A}_{pu} \sin \Omega t}{\Omega}\right]\right\} |\psi^{v}\rangle \\ &= \sum_{n=-\infty}^{\infty} J_n\left(\frac{\mathbf{v}(\mathbf{k}_{\|}) \cdot \mathbf{A}_{pu}}{\hbar\Omega}\right) \exp\left\{-\frac{i}{\hbar}\left[\hat{H}_{\Gamma}(\mathbf{k}_{\|}) + n\hbar\Omega\right] t\right\} |\psi^{v}\rangle\end{aligned} \tag{S8}$$

Herein, we have used the Jacobi-Anger relation $e^{iz\sin\theta} = \sum_{n=-\infty}^{\infty} J_n(z) e^{in\theta}$, where $J_n(z)$ is the n-th Bessel function of the first kind. This solution reflects the structures of the Floquet state distinctly (*13*):

$$\begin{aligned}\left|\Psi^{v}\left(t, \mathbf{k}_{\|}\right)\right\rangle &= \sum_{n=-\infty}^{\infty} J_n\left(\frac{\mathbf{v}(\mathbf{k}_{\|}) \cdot \mathbf{A}_{pu}}{\hbar\Omega}\right) \exp\left\{-\frac{i}{\hbar}\left[\hat{H}_{\Gamma}(\mathbf{k}_{\|}) + n\hbar\Omega\right] t\right\} |\psi^{v}\rangle \\ &= \exp\left(-\frac{i}{\hbar}\hat{H}_{\Gamma}(\mathbf{k}_{\|})t\right) \sum_{n=-\infty}^{\infty} e^{-in\Omega t}\left[J_n\left(\frac{\mathbf{v}(\mathbf{k}_{\|}) \cdot \mathbf{A}_{pu}}{\hbar\Omega}\right) |\psi^{v}\rangle\right] \\ &= \exp\left(-\frac{i}{\hbar}\hat{H}_{\Gamma}(\mathbf{k}_{\|})t\right) \sum_{n=-\infty}^{\infty} e^{-in\Omega t} \left|\psi_n^{v}\right\rangle\end{aligned} \tag{S9}$$

Here, $\left|\psi_n^{v}\right\rangle$ possesses the weight $J_n\left(\frac{\mathbf{v}(\mathbf{k}_{\|}) \cdot \mathbf{A}_{pu}}{\hbar\Omega}\right)$ (*64*) and the Floquet quasi-energy is $\hat{H}_{\Gamma}(\mathbf{k}_{\|}) + n\hbar\Omega$.

Moreover, we focus on the spectra around the Γ point ($\mathbf{k}_{\|} \rightarrow 0$), namely $\left(\frac{\mathbf{v}(\mathbf{k}_{\|}) \cdot \mathbf{A}_{pu}}{\hbar\Omega}\right) \rightarrow 0$, then we can employ the asymptotic expansion of the Bessel function of the first kind when $z \rightarrow 0$:

$$J_n(z) \simeq \frac{1}{\Gamma(n+1)}\left(\frac{z}{2}\right)^n + O(z^{n+2}) \tag{S10}$$

where $\Gamma(n+1) = \int_0^{+\infty} e^{-t} t^n dt$ is the Gamma function. For $n \geq 0$, we can further simplify $\left|\psi_n^v\right\rangle$ in Eq. S9 as

$$\begin{aligned}\left|\psi_n^v\right\rangle =& J_n\left(\frac{\mathbf{v}(\mathbf{k}_{\|}) \cdot \mathbf{A}_{pu}}{\hbar\Omega}\right)\left|\psi^v\right\rangle \\ \simeq& \frac{1}{\Gamma(n+1)}\left(\frac{\mathbf{v}(\mathbf{k}_{\|}) \cdot \mathbf{A}_{pu}}{2\hbar\Omega}\right)^n\left|\psi^v\right\rangle\end{aligned} \tag{S11}$$

We notice that, $\mathbf{v}(\mathbf{k}_{\|}) \cdot \mathbf{A}_{pu}$ is just the light-matter interaction Hamiltonian for the pumping laser. For $n < 0$, considering $J_{-n}(z) = (-1)^n J_n(z)$, we can also simplify $\left|\psi_n^v\right\rangle$ in Eq. S9 as

$$\begin{aligned}\left|\psi_n^v\right\rangle =& J_n\left(\frac{\mathbf{v}(\mathbf{k}_{\|}) \cdot \mathbf{A}_{pu}}{\hbar\Omega}\right)\left|\psi^v\right\rangle \\ =& (-1)^n J_{-n}\left(\frac{\mathbf{v}(\mathbf{k}_{\|}) \cdot \mathbf{A}_{pu}}{\hbar\Omega}\right)\left|\psi^v\right\rangle \\ \simeq& \frac{(-1)^n}{\Gamma(1-n)}\left(\frac{\mathbf{v}(\mathbf{k}_{\|}) \cdot \mathbf{A}_{pu}}{2\hbar\Omega}\right)^{-n}\left|\psi^v\right\rangle\end{aligned} \tag{S12}$$

So in general the representation of $\left|\psi_n^v\right\rangle$ is just the representation of the term $(\mathbf{v}(\mathbf{k}_{\|}) \cdot \mathbf{A}_{pu})^{|n|}\left|\psi^v\right\rangle$.

Floquet-Volkov states — Furthermore, if the pumping laser field possesses an out-of-plane component $A_{pu}^z(t) = A_{pu}^z \cos\Omega t$, it is necessary for us to account for the interference between the Floquet and Volkov states (*14*) around the Γ point. Consequently, the wavefunction of the n-th sideband for the VB in Eq. S9 can be written as

$$\left|\psi_n^v\right\rangle = J_n\left(\frac{\mathbf{v}(\mathbf{k}_{\|}) \cdot \mathbf{A}_{pu} + v_z A_{pu}^z}{\hbar\Omega}\right)\left|\psi^v\right\rangle \tag{S13}$$

where v_z denotes the out-of-plane velocity of the emitted electron (*13*). Though $\left(\mathbf{v}(\mathbf{k}_{\|}) \cdot \mathbf{A}_{pu} + v_z A_{pu}^z\right)$ doesn't approach zero around the Γ point, we can still conduct a more general analysis on Eq. S13. Utilizing the series expansion of the n-th Bessel function of the first kind, we proceed to expand Eq. S13 for $n \geq 0$ as

$$\begin{aligned}\left|\psi_n^v\right\rangle =& J_n\left(\frac{\mathbf{v}(\mathbf{k}_{\|}) \cdot \mathbf{A}_{pu} + v_z A_{pu}^z}{\hbar\Omega}\right)\left|\psi^v\right\rangle \\ =& \sum_{k=0}^{\infty} \frac{(-1)^k}{k!\Gamma(k+n+1)}\left(\frac{\mathbf{v}(\mathbf{k}_{\|}) \cdot \mathbf{A}_{pu} + v_z A_{pu}^z}{2\hbar\Omega}\right)^{2k+n}\left|\psi^v\right\rangle \\ =& \left[\sum_{k=0}^{\infty} \frac{(-1)^k}{k!\Gamma(k+n+1)}\left(\frac{\mathbf{v}(\mathbf{k}_{\|}) \cdot \mathbf{A}_{pu} + v_z A_{pu}^z}{2\hbar\Omega}\right)^{2k}\right]\left[\left(\frac{\mathbf{v}(\mathbf{k}_{\|}) \cdot \mathbf{A}_{pu} + v_z A_{pu}^z}{2\hbar\Omega}\right)^n\left|\psi^v\right\rangle\right]\end{aligned} \tag{S14}$$

Similarly, for $n < 0$, we obtain

$$
\begin{aligned}
\left|\psi_n^v\right\rangle =& J_n\left(\frac{\mathbf{v}(\mathbf{k}_{\|})\cdot\mathbf{A}_{pu}+v_zA_{pu}^z}{\hbar\Omega}\right)\left|\psi^v\right\rangle \\
=& \sum_{k=0}^{\infty}\frac{(-1)^{k+n}}{k!\Gamma(k-n+1)}\left(\frac{\mathbf{v}(\mathbf{k}_{\|})\cdot\mathbf{A}_{pu}+v_zA_{pu}^z}{2\hbar\Omega}\right)^{2k-n}\left|\psi^v\right\rangle \\
=& \left[\sum_{k=0}^{\infty}\frac{(-1)^{k+n}}{k!\Gamma(k-n+1)}\left(\frac{\mathbf{v}(\mathbf{k}_{\|})\cdot\mathbf{A}_{pu}+v_zA_{pu}^z}{2\hbar\Omega}\right)^{2k}\right]\left[\left(\frac{\mathbf{v}(\mathbf{k}_{\|})\cdot\mathbf{A}_{pu}+v_zA_{pu}^z}{2\hbar\Omega}\right)^{-n}\left|\psi^v\right\rangle\right]
\end{aligned}
\tag{S15}
$$

So the representation of $\left|\psi_n^v\right\rangle$ is also determined by the representation of $(\mathbf{v}(\mathbf{k}_{\|})\cdot\mathbf{A}_{pu} + v_zA_{pu}^z)^{|n|}\left|\psi^v\right\rangle$. Next we take several cases as examples to analyze the direct product among the representations of $\hat{H}'$ and $\left|\psi_n^v\right\rangle$.

Case 1 — AC (s-$pol.$)@AC (s-$pol.$):

The polarization directions of the pumping and probe laser are both totally along the AC direction. So now $(\mathbf{v}(\mathbf{k}_{\|})\cdot\mathbf{A}_{pu})^{|n|} \simeq (k_xA_{pu}^x)^{|n|}$, whose representation is $(\Gamma_3^-)^{|n|}$. Then we obtain the representation of $\hat{H}'|\psi_n^v\rangle$ as

$$
\underbrace{\Gamma_3^-}_{probe}\otimes(\underbrace{\Gamma_3^-}_{pump})^{|n|}\otimes\underbrace{\Gamma_2^+}_{|\psi^v\rangle} = (\Gamma_3^-)^{|n|+1}\otimes\Gamma_2^+
\tag{S16}
$$

1. n is **even**, then $(\Gamma_3^-)^{|n|+1}\otimes\Gamma_2^+ = \Gamma_1^+\otimes\Gamma_3^-\otimes\Gamma_2^+ = \Gamma_4^-$, which is also **even**. So around the Γ point, the n-th sideband for the VB can be detected under the AC (s-$pol.$) probe laser.
2. n is **odd**, then $(\Gamma_3^-)^{|n|+1}\otimes\Gamma_2^+ = \Gamma_1^+\otimes\Gamma_2^+ = \Gamma_2^+$, which is also **odd**. So around the Γ point, the n-th sideband for the VB can't be detected under the AC (s-$pol.$) probe laser.

Case 2 — AC (s-$pol.$)@ZZ (p-$pol.$):

Similar to the Case 1 but the polarization direction of the probe laser is in the scattering plane. In the same way we obtain the representation as: $(\Gamma_2^-\oplus\Gamma_4^-)\otimes(\Gamma_3^-)^{|n|}\otimes\Gamma_2^+$

1. n is **even**, then $(\Gamma_2^-\oplus\Gamma_4^-)\otimes(\Gamma_3^-)^{|n|}\otimes\Gamma_2^+ = (\Gamma_2^-\oplus\Gamma_4^-)\otimes\Gamma_1^+\otimes\Gamma_2^+ = \Gamma_1^-\oplus\Gamma_3^-$, which is **odd**, namely, the n-th sideband for the VB can't be detected under the ZZ (p-$pol.$) probe laser around the Γ point.

2. n is **odd**, then $(\Gamma_2^- \oplus \Gamma_4^-) \otimes (\Gamma_3^-)^{|n|} \otimes \Gamma_2^+ = (\Gamma_2^- \oplus \Gamma_4^-) \otimes \Gamma_3^- \otimes \Gamma_2^+ = (\Gamma_2^- \oplus \Gamma_4^-) \otimes \Gamma_4^- = \Gamma_3^+ \oplus \Gamma_1^+$, which is **even**, namely, the n-th sideband for the VB can be detected under the ZZ (p-$pol.$) probe laser around the Γ point.

Case 3 — ZZ (p-$pol.$)@AC (s-$pol.$):

The polarization direction of the pumping laser is in the scattering plane. So now $(\mathbf{v}(\mathbf{k}_{||}) \cdot \mathbf{A}_{pu} + v_z A_{pu}^z)^{|n|} \simeq (k_y A_{pu}^y + v_z A_{pu}^z)^{|n|}$, whose representation is $(\Gamma_2^- \oplus \Gamma_4^-)^{|n|}$. Then we obtain: $\Gamma_3^- \otimes (\Gamma_2^- \oplus \Gamma_4^-)^{|n|} \otimes \Gamma_2^+ = (\Gamma_2^- \oplus \Gamma_4^-)^{|n|} \otimes \Gamma_4^- = C_{|n|}^r (\Gamma_2^-)^r \otimes (\Gamma_4^-)^{|n|-r+1}$, this term must be **even**, indicating the n-th sideband for the VB can be detected under the AC (s-$pol.$) probe laser around the Γ point.

Case 4 — ZZ (p-$pol.$)@ZZ (p-$pol.$):

Similar to the Case 3 but the polarization direction of the probe laser is also in the scattering plane. So in the same way we obtain: $(\Gamma_2^- \oplus \Gamma_4^-) \otimes (\Gamma_2^- \oplus \Gamma_4^-)^{|n|} \otimes \Gamma_2^+ = (\Gamma_2^- \oplus \Gamma_4^-)^{|n|} \otimes (\Gamma_1^- \oplus \Gamma_3^-)$, this term must be **odd**, indicating the n-th sideband for the VB can't be detected under the AC (s-$pol.$) probe laser around the Γ point.

In summary, we list symmetry analyses of the matrix element $\mathcal{M}$ under different pump-probe conditions in Table S4.

Table S4: The symmetry analysis of the TrARPES matrix element.

	Pump laser		Probe laser		$\hat{H}'\|\psi_n^v\rangle$		Experimental evidence in references
	Polarization direction	Group representation	Polarization direction	Group representation	Group representation	Symmetry	
1	AC (s-$pol.$)	Γ_3^-	AC (s-$pol.$)	Γ_3^-	$\Gamma_3^- \otimes (\Gamma_3^-)^{\|n\|} \otimes \Gamma_2^+$	even (n is even)<br>odd (n is odd)	(*38*), Fig. 4A<br>(*39*), Fig. 2C
2	AC (s-$pol.$)	Γ_3^-	ZZ (p-$pol.$)	$\Gamma_2^- \oplus \Gamma_4^-$	$(\Gamma_2^- \oplus \Gamma_4^-) \otimes (\Gamma_3^-)^{\|n\|} \otimes \Gamma_2^+$	odd (n is even)<br>even (n is odd)	(*44*), Fig. 2G
3	ZZ (p-$pol.$)	$\Gamma_2^- \oplus \Gamma_4^-$	AC (s-$pol.$)	Γ_3^-	$\Gamma_3^- \otimes (\Gamma_2^- \oplus \Gamma_4^-)^{\|n\|} \otimes \Gamma_2^+$	even	(*38*), Fig. 4B<br>(*39*), Fig. S1F
4	ZZ (p-$pol.$)	$\Gamma_2^- \oplus \Gamma_4^-$	ZZ (p-$pol.$)	$\Gamma_2^- \oplus \Gamma_4^-$	$(\Gamma_2^- \oplus \Gamma_4^-) \otimes (\Gamma_2^- \oplus \Gamma_4^-)^{\|n\|} \otimes \Gamma_2^+$	odd	——

3 Supplementary Note 3: TrARPES intensity plots

In this section, we show some TDDFT-simulated TrARPES intensity plots and energy distribution curves (EDCs) of monolayer BP without any doping under different pump-probe conditions.

3.1 ZZ (p-$pol.$)@AC (s-$pol.$) configuration

To investigate the temporal dynamics of Floquet-Volkov states under the experimental setup depicted in the inset of Fig. S3A, we vary the pump-probe delay time Δt to -50 fs, -10 fs, 0 fs, 10 fs, 20 fs, 100 fs, respectively, to simulate the TrARPES intensity plots, as shown in Fig. S3.

Moreover, we extract EDCs at the Γ point from Fig. S3 under different pump-probe delay times, as shown in Fig. S4A. The energy interval between adjacent peaks, corresponding to the pump photon energy, signifies the presence of sidebands. Inset 1 in Fig. S4A shows that the energy position of the n=0 sideband peak remains nearly unchanged across different delay times, implying no energy shift around the Γ point. As the delay time increases, the reduced temporal overlap between the pumping and probe pulses results in an intensity increase for the n=0 sideband (see inset 1), while a decrease for sidebands with $n \neq 0$ (see inset 2 in Fig. S4A) and eventually dissolution completely, indicating the formation and collapse for Floquet-Volkov states (*12*).

To visually depict this behavior, we calculate spectral weights based on Fig. S4A, as presented in Fig. S4B. At a delay time of -50 fs, the $n = -1$ sideband begins to emerge. At 0 fs, when the pumping and probe pulses are in complete overlap, the spectral weights are largest for the sidebands with $n \neq 0$ and smallest for the n=0 sideband. With the delay time increasing, the spectral weights of the $n \neq 0$ sidebands gradually diminish, while the n=0 sideband becomes stronger. Our TDDFT simulations succeed in visualizing the evolution of Floquet-Volkov states in the time domain, which has been observed in TrARPES measurements (*38*).

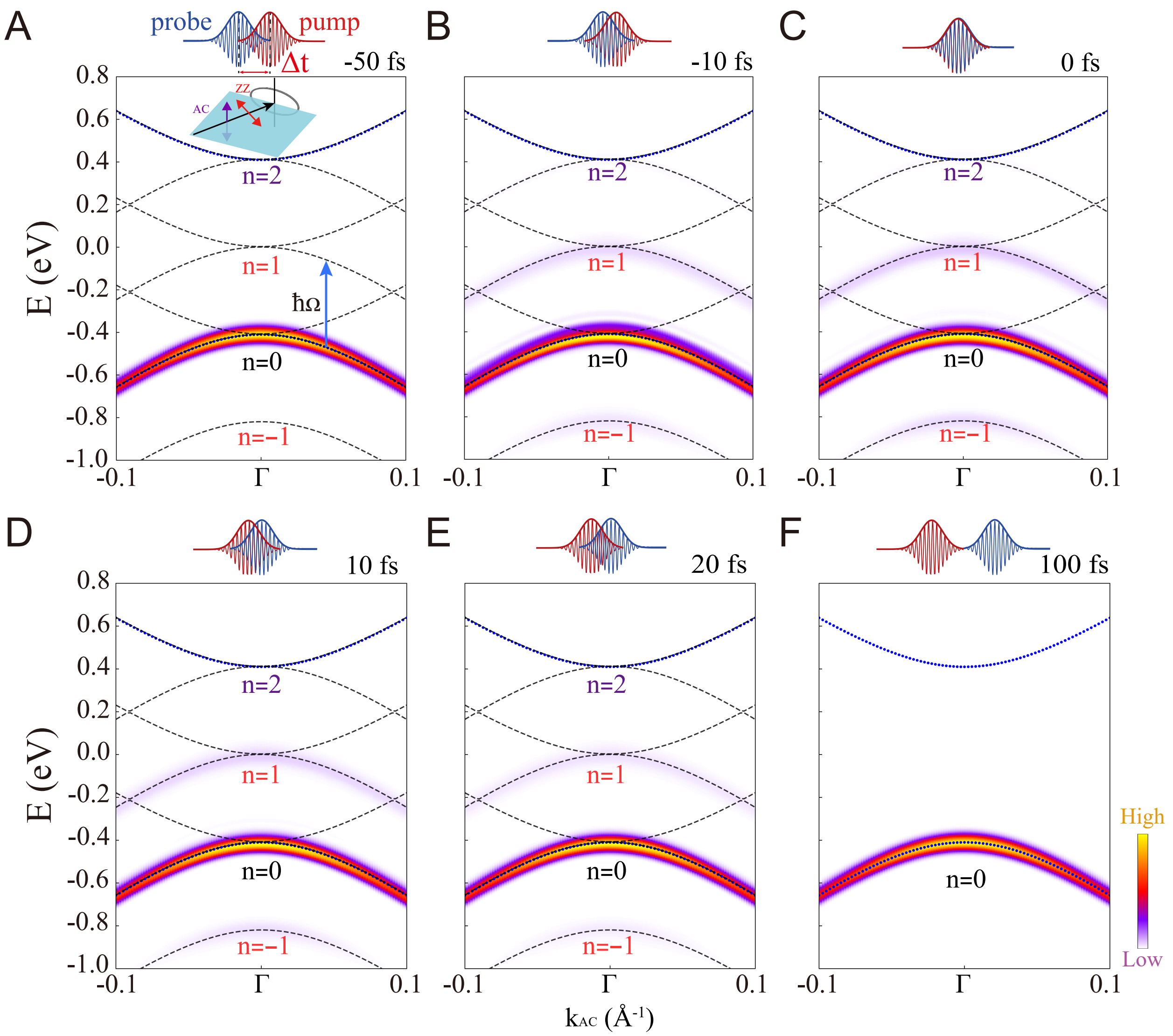

Figure S3: The simulated TrARPES intensity plots [ZZ (p-$pol.$)@AC (s-$pol.$)] under different pump-probe delay time. (**A**) The TrARPES intensity plot calculated along the AC direction upon ZZ (p-$pol.$) pumping laser. The polarization of the probe laser is along the AC (s-$pol.$) direction, as depicted in the inset. The pump-probe delay time Δt is set to -50 fs, and the peak intensity I_{pu} of the pumping laser is 1×10^{10} W/cm^2, and all other parameters remain consistent with the below-gap pumping case in the main text. (**B-F**) Similar results to (A) but the pump-probe delay time Δt is adjusted to -10 fs, 0 fs, 10 fs, 20 fs, and 100 fs, respectively. The blue dot lines are equilibrium band structures, and the black dashed lines are light-induced band structures for these spectra.

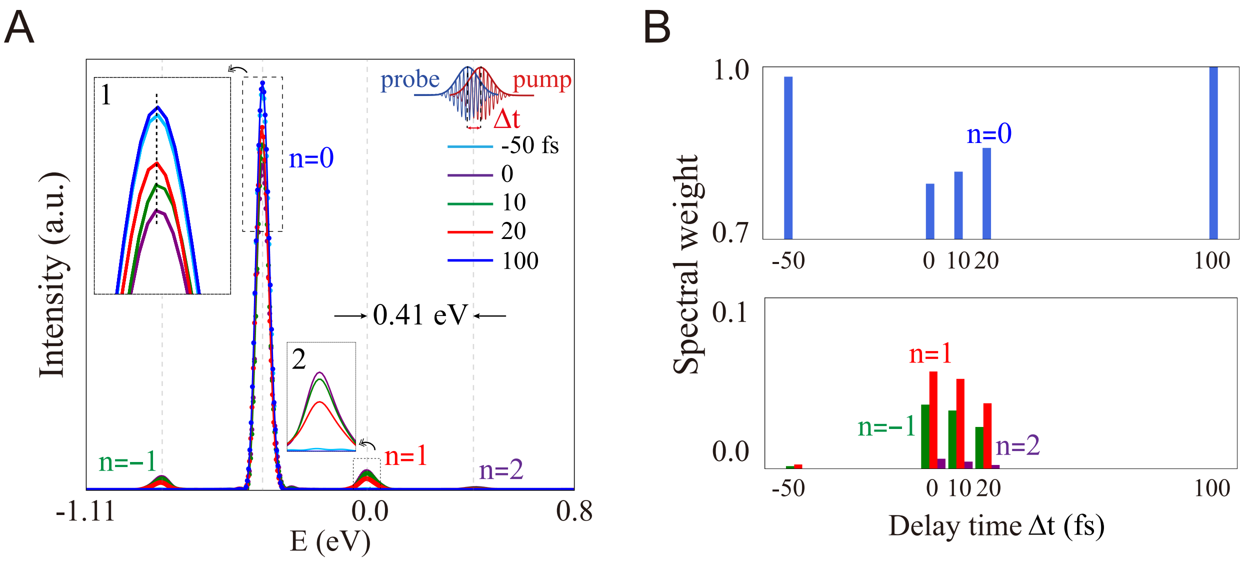

Figure S4: The analysis of extracted EDCs and spectral weights. (**A**) EDCs extracted at the Γ point under different pump-probe delay times. (**B**) Extracted spectral weight of different sidebands at different delay times from EDCs in (A).

3.2 AC (s-$pol.$)@ZZ (p-$pol.$) configuration

In this section, we systematically investigate the AC (s-$pol.$)@ZZ (p-$pol.$) configuration by examining the spectra under varying the pump-probe delay time Δt (Fig. S5), the peak intensity I_{pu} of the pumping laser (Fig. S6) and the pulse duration time (Fig. S7). Remarkably, across all parameter variations, the characteristic spot-like spectral signature of the n=1 Floquet VB persists, demonstrating the robustness of this feature. This persistence directly reflects the underlying symmetry constraints imposed by the Floquet optical selection rules, which remain invariant under these parameter changes while maintaining the fixed pump-probe configuration.

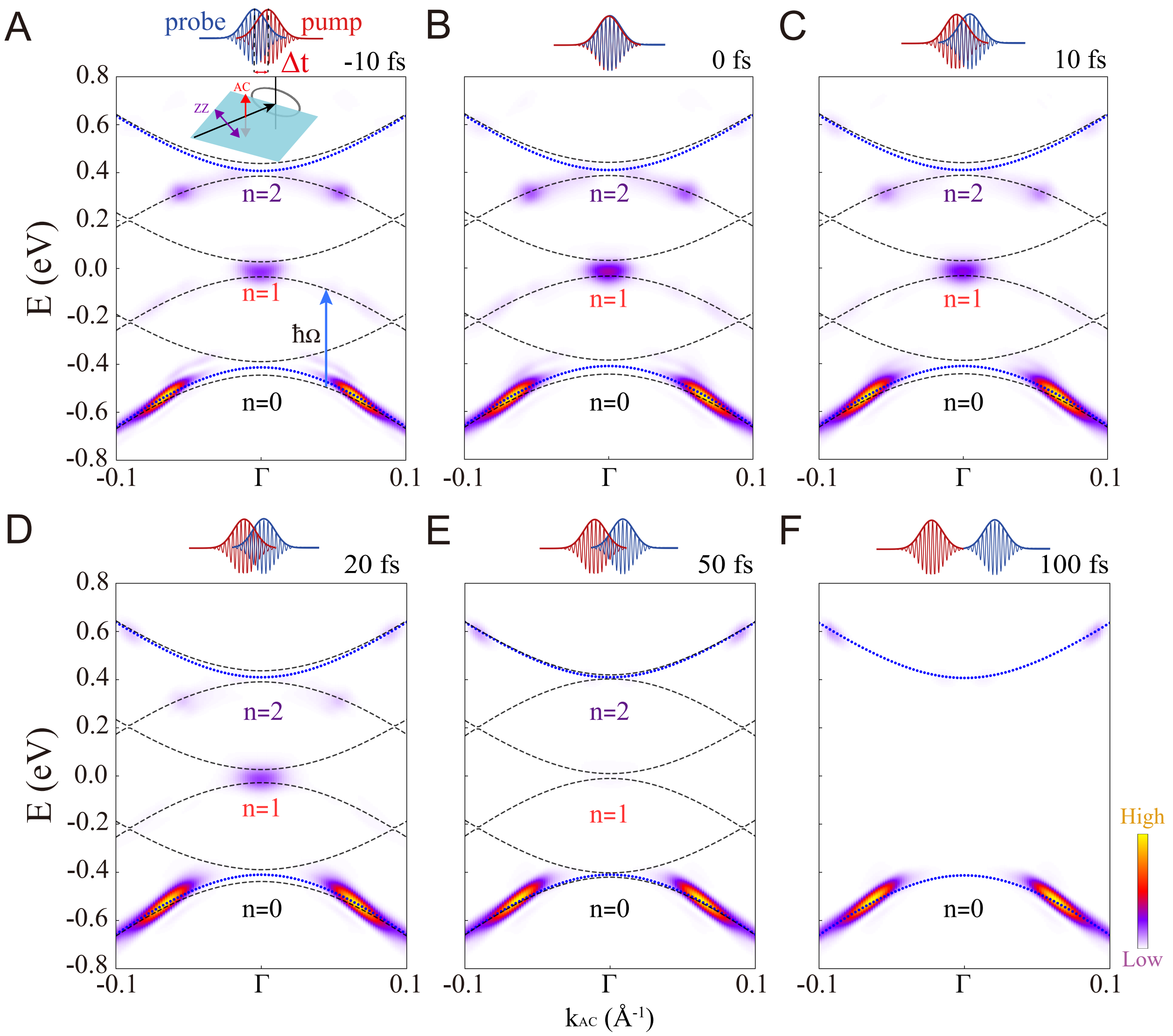

Figure S5: The simulated TrARPES intensity plots [AC (s-$pol.$)@ZZ (p-$pol.$)] under different pump-probe delay time. (**A**) The TrARPES intensity plot calculated along the AC direction upon AC (s-$pol.$) pumping laser. The polarization of the probe laser is along the ZZ (p-$pol.$) direction, as depicted in the inset. The pump-probe delay time Δt is set to -10 fs, and all other parameters remain consistent with the below-gap pumping case in the main text. (**B-F**) Similar results to (A) but the pump-probe delay time Δt is adjusted to 0 fs, 10 fs, 20 fs, 50 fs, and 100 fs, respectively. The blue dot lines are equilibrium band structures, and the black dashed lines are Floquet band structures for these spectra.

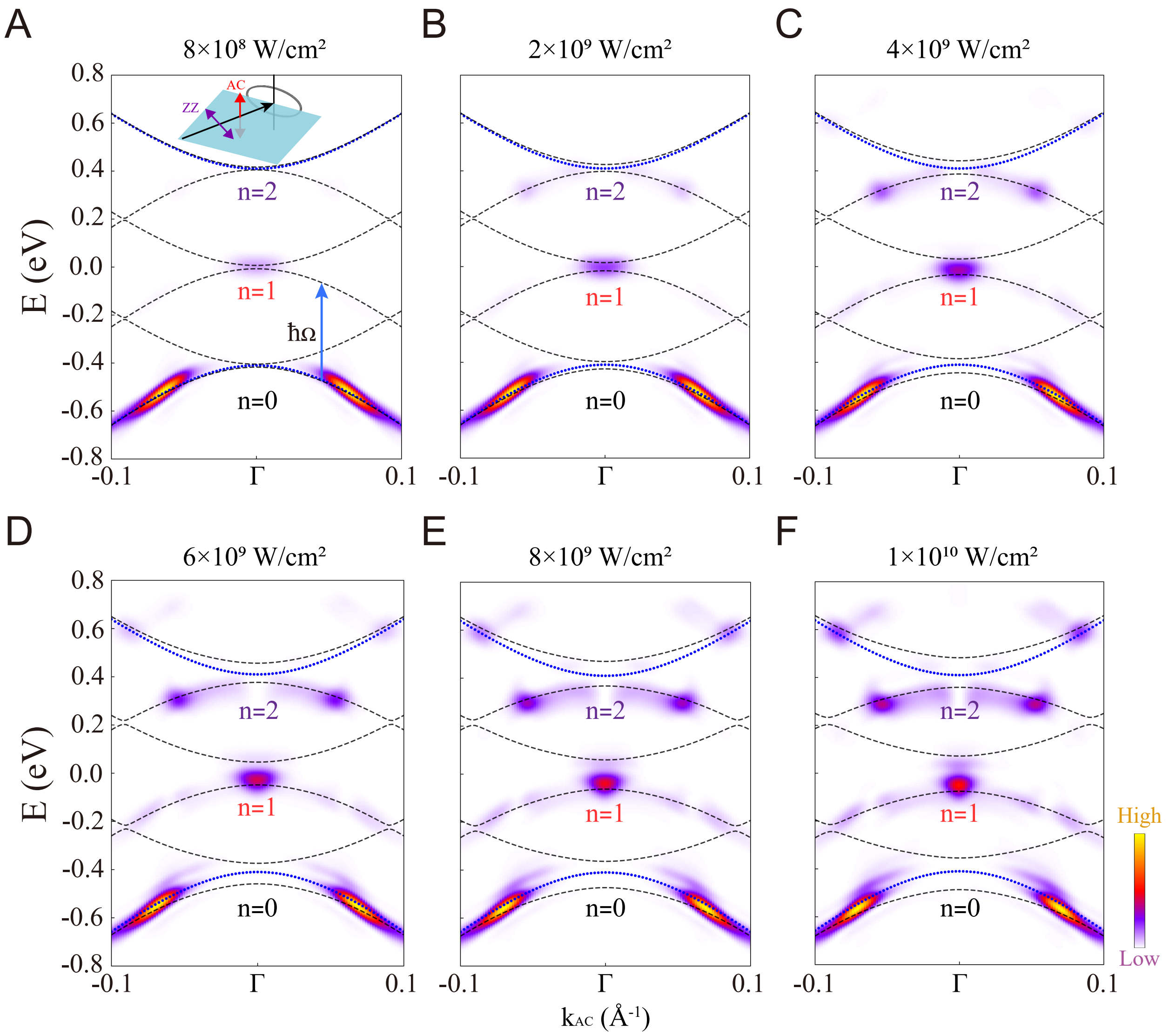

Figure S6: The simulated TrARPES intensity plots [AC (s-$pol.$)@ZZ (p-$pol.$)] under different peak intensity I_{pu} of the pumping laser. (**A**) The TrARPES intensity plot calculated along the AC direction upon AC (s-$pol.$) pumping laser. The polarization of the probe laser is along the ZZ (p-$pol.$) direction, as depicted in the inset. The peak intensity I_{pu} of the pumping laser is set to 8×10^8 W/cm^2, and all other parameters remain consistent with the below-gap pumping case in the main text. (**B-F**) Similar results to (A) but the peak intensity I_{pu} of the pumping laser is adjusted to 2×10^9 W/cm^2, 4×10^9 W/cm^2, 6×10^9 W/cm^2, 8×10^9 W/cm^2, and 1×10^{10} W/cm^2, respectively. The blue dot lines are equilibrium band structures, and the black dashed lines are Floquet band structures for these spectra.

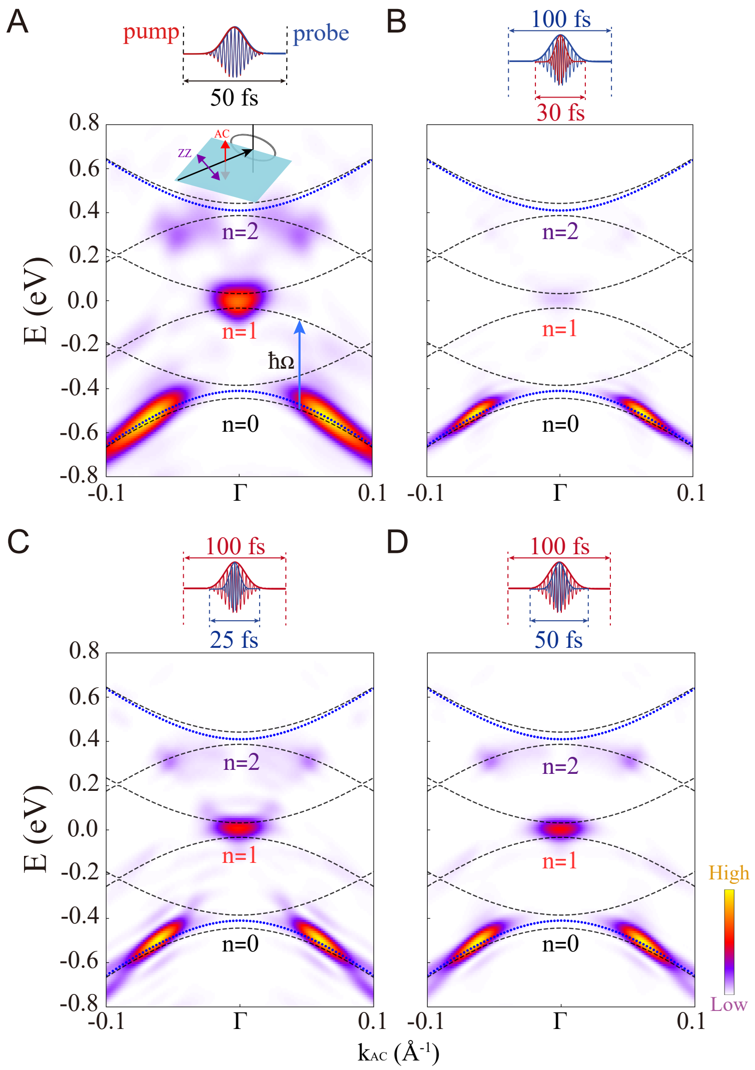

Figure S7: The simulated TrARPES intensity plots [AC (s-$pol.$)@ZZ (p-$pol.$)] under different pulse duration time. (**A**) The TrARPES intensity plot calculated along the AC direction upon AC (s-$pol.$) pumping laser. The polarization of the probe laser is along the ZZ (p-$pol.$) direction, as depicted in the inset. The pulse duration T_{pu} of the pumping laser is set to 50 fs, matching the pulse duration T_{pr} of the probe laser, and all other parameters remain consistent with the below-gap pumping case in the main text. (**B**) Similar result to (A), but with the pulse duration T_{pu} set to 30 fs and T_{pr} set to 100 fs. (**C**) Similar result to (B), but with the pulse duration T_{pu} set to 100 fs and T_{pr} set to 25 fs. (**D**) Similar result to (C), but with the pulse duration T_{pr} set to 50 fs. The blue dot lines are equilibrium band structures, and the black dashed lines are Floquet band structures for these spectra. The pump-probe delay time Δt is set to 0 fs.

4 Supplementary Note 4: A case study of monolayer hexagonal boron nitride (*h*-BN)

In this section, we further take the monolayer *h*-BN as an example to investigate its spectral features of the light-induced sidebands around the K point, where the band structures are also approximately parabolic.

At the K point, the point group of *h*-BN is C_{3h}, which is an Abelian group with a mirror symmetry M_z (denotes as M in Table S5). For the VB wavefunction $|\psi^v\rangle$ around the K point, it is mainly contributed by the p_z orbitals, corresponding to group representation Γ_4. Next, we choose the polarization directions of the pumping laser in the xy plane (*h*-BN plane) and the probe laser along the z direction. Similarly, we can obtain the group representation of $\hat{H}'|\psi_n^v\rangle$ at the K point as

$$\underbrace{\Gamma_4}_{probe} \otimes \underbrace{(\Gamma_2 \oplus \Gamma_3)^{|n|}}_{pump} \otimes \underbrace{\Gamma_4}_{|\psi^v\rangle} = (\Gamma_2 \oplus \Gamma_3)^{|n|} \tag{S17}$$

Since Γ_2 and Γ_3 share the same character 1 and the character of Γ_4 is -1 under the mirror operation M in the C_{3h} group, the resulting overall group representation in Eq. S17 is always even, regardless of n being even or odd. This implies that all Floquet-Volkov sidebands with n=0, ±1 for the VB around the K point are symmetry-allowed and should be observable in TrARPES. As shown in Fig. S8, our TDDFT simulations of the TrARPES intensity plot for the monolayer *h*-BN along the Γ−K−Γ path confirm this prediction, showing clear spectral features around the K point pointed by red and blue arrows, in agreement with our group-theoretical analysis.

Table S5: The character table for the point group C_{3h}.

	E	C_3	C_3^2	M	S_3	MC_3^2	Basis
Γ_1	1	1	1	1	1	1	x^2+y^2, z^2, J_z
Γ_4	1	1	1	-1	-1	-1	z
Γ_2	1	w	w^2	1	w	w^2	$(x, y), (x^2-y^2, xy)$
Γ_3	1	w^2	w	1	w^2	w	
Γ_5	1	w	w^2	-1	$-w$	$-w^2$	$(xz, yz), (J_x, J_y)$
Γ_6	1	w^2	w	-1	$-w^2$	$-w$	

$w = e^{2\pi i/3}$

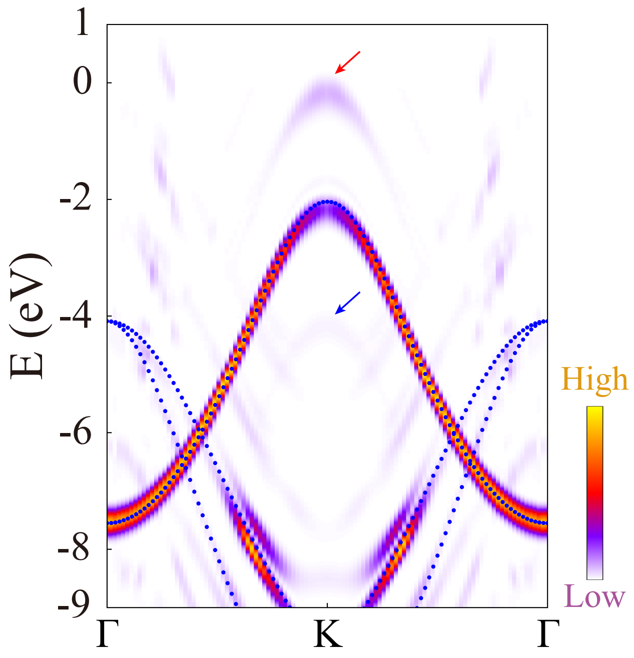

Figure S8: The simulated TrARPES intensity plot along the Γ−K−Γ direction under below-gap pumping for *h*-BN. For the pumping laser, the photon energy $\hbar\Omega$ is 2 eV and the peak intensity I_{pu} is 2×10^{12} W/cm^2 with a pulse duration T_{pu} of 18 fs. For the probe laser, the photon energy $\hbar\omega$ is 40.8 eV and the peak intensity I_{pr} is 1×10^8 W/cm^2 with a pulse duration T_{pr} of 18 fs. The pump-probe delay time Δt is set to 0 fs. The blue dot lines are equilibrium band structures. The red (blue) arrow points to the n=1 (n=−1) light-induced sideband around the K point.

REFERENCES AND NOTES

1. D. N. Basov, R. D. Averitt, D. Hsieh, Towards properties on demand in quantum materials. *Nat. Mater.* **16**, 1077–1088 (2017).

2. A. de la Torre, D. M. Kennes, M. Claassen, S. Gerber, J. W. McIver, M. A. Sentef, Colloquium: Nonthermal pathways to ultrafast control in quantum materials. *Rev. Mod. Phys.* **93**, 041002 (2021).

3. J. Qiao, X. Kong, Z.-X. Hu, F. Yang, W. Ji, High-mobility transport anisotropy and linear dichroism in few-layer black phosphorus. *Nat. Commun.* **5**, 4475 (2014).

4. H. Yuan, X. Liu, F. Afshinmanesh, W. Li, G. Xu, J. Sun, B. Lian, A. G. Curto, G. Ye, Y. Hikita, Z. Shen, S.-C. Zhang, X. Chen, M. Brongersma, H. Y. Hwang, Y. Cui, Polarization-sensitive broadband photodetector using a black phosphorus vertical p–n junction. *Nat. Nanotechnol.* **10**, 707–713 (2015).

5. K. F. Mak, K. He, J. Shan, T. F. Heinz, Control of valley polarization in monolayer MoS_2 by optical helicity. *Nat. Nanotechnol.* **7**, 494–498 (2012).

6. H. Zeng, J. Dai, W. Yao, D. Xiao, X. Cui, Valley polarization in MoS2 monolayers by optical pumping. *Nat. Nanotechnol.* **7**, 490–493 (2012).

7. J. Bloch, A. Cavalleri, V. Galitski, M. Hafezi, A. Rubio, Strongly correlated electron–photon systems. *Nature* **606**, 41–48 (2022).

8. C. Bao, P. Tang, D. Sun, S. Zhou, Light-induced emergent phenomena in 2D materials and topological materials. *Nat. Rev. Phys.* **4**, 33–48 (2022).

9. Y. Wang, H. Steinberg, P. Jarillo-Herrero, N. Gedik, Observation of Floquet-Bloch states on the surface of a topological insulator. *Science* **342**, 453–457 (2013).

10. S. Aeschlimann, S. A. Sato, R. Krause, M. Chávez-Cervantes, U. de Giovannini, H. Hübener, S. Forti, C. Coletti, K. Hanff, K. Rossnagel, A. Rubio, I. Gierz, Survival of Floquet-Bloch states in the presence of scattering. *Nano Lett.* **21**, 5028–5035 (2021).

11. S. Park, W. Lee, S. Jang, Y. B. Choi, J. Park, W. Jung, K. Watanabe, T. Taniguchi, G. Y. Cho, G. H. Lee, Steady Floquet-Andreev states in graphene Josephson junctions. *Nature* **603**, 421–426 (2022).

12. S. Ito, M. Schüler, M. Meierhofer, S. Schlauderer, J. Freudenstein, J. Reimann, D. Afanasiev, K. A. Kokh, O. E. Tereshchenko, J. Güdde, M. A. Sentef, U. Höfer, R. Huber, Build-up and dephasing of Floquet–Bloch bands on subcycle timescales. *Nature* **616**, 696–701 (2023).

13. S. T. Park, Interference in Floquet-Volkov transitions. *Phys. Rev. A* **90**, 013420 (2014).

14. F. Mahmood, C.-K. Chan, Z. Alpichshev, D. Gardner, Y. Lee, P. A. Lee, N. Gedik, Selective scattering between Floquet–Bloch and Volkov states in a topological insulator. *Nat. Phys.* **12**, 306–310 (2016).

15. D. Choi, M. Mogi, U. De Giovannini, D. Azoury, B. Lv, Y. Su, H. Hübener, A. Rubio, N. Gedik, Observation of Floquet–Bloch states in monolayer graphene. *Nat. Phys.* **21**, 1100–1105 (2025).

16. M. Merboldt, M. Schüler, D. Schmitt, J. P. Bange, W. Bennecke, K. Gadge, K. Pierz, H. W. Schumacher, D. Momeni, D. Steil, S. R. Manmana, M. A. Sentef, M. Reutzel, S. Mathias, Observation of Floquet states in graphene. *Nat. Phys.* **21**, 1093–1099 (2025).

17. T. Oka, H. Aoki, Photovoltaic Hall effect in graphene. *Phys. Rev. B* **79**, 081406 (2009).

18. M. S. Rudner, N. H. Lindner, Band structure engineering and non-equilibrium dynamics in Floquet topological insulators. *Nat. Rev. Phys.* **2**, 229–244 (2020).

19. N. H. Lindner, G. Refael, V. Galitski, Floquet topological insulator in semiconductor quantum wells. *Nat. Phys.* **7**, 490–495 (2011).

20. M. Sentef, M. Claassen, A. F. Kemper, B. Moritz, T. Oka, J. K. Freericks, T. P. Devereaux, Theory of Floquet band formation and local pseudospin textures in pumpprobe photoemission of graphene. *Nat. Commun.* **6**, 7047 (2015).

21. C.-K. Chan, P. A. Lee, K. S. Burch, J. H. Han, Y. Ran, When chiral photons meet chiral fermions: Photoinduced anomalous Hall effects in Weyl semimetals. *Phys. Rev. Lett.* **116**, 026805 (2016).

22. Z. Yan, Z. Wang, Tunable Weyl points in periodically driven nodal line semimetals. *Phys. Rev. Lett.* **117**, 087402 (2016).

23. M. Claassen, C. Jia, B. Moritz, T. P. Devereaux, All-optical materials design of chiral edge modes in transition-metal dichalcogenides. *Nat. Commun.* **7**, 13074 (2016).

24. U. De Giovannini, H. Hubener, A. Rubio, Monitoring electron-photon dressing in WSe_2. *Nano Lett.* **16**, 7993–7998 (2016).

25. H. Hübener, M. A. Sentef, U. De Giovannini, A. F. Kemper, A. Rubio, Creating stable Floquet-Weyl semimetals by laser-driving of 3D Dirac materials. *Nat. Commun.* **8**, 13940 (2017).

26. H. Liu, J.-T. Sun, C. Cheng, F. Liu, S. Meng, Photoinduced nonequilibrium topological states in strained black phosphorus. *Phys. Rev. Lett.* **120**, 237403 (2018).

27. S. Sato, J. W. Mc Iver, M. Nuske, P. Tang, G. Jotzu, B. Schulte, H. Hübener, U. De Giovannini, L. Mathey, M. A. Sentef, A. Cavalleri, A. Rubio, Microscopic theory for the light-induced anomalous Hall effect in graphene. *Phys. Rev. B* **99**, 214302 (2019).

28. J. W. McIver, B. Schulte, F. U. Stein, T. Matsuyama, G. Jotzu, G. Meier, A. Cavalleri, Light-induced anomalous Hall effect in graphene. *Nat. Phys.* **16**, 38–41 (2020).

29. O. Neufeld, H. Hubener, G. Jotzu, U. De Giovannini, A. Rubio, Band nonlinearity-enabled manipulation of Dirac nodes, Weyl cones, and valleytronics with intense linearly polarized light. *Nano Lett.* **23**, 7568–7575 (2023).

30. T. Zhu, H. Wang, H. Zhang, Floquet engineering of magnetic topological insulator $MnBi_2Te_4$ films. *Phys. Rev. B* **107**, 085151 (2023).

31. X. Liu, B. Fan, H. Hübener, U. De Giovannini, W. Duan, A. Rubio, P. Tang, Floquet engineering of magnetism in topological insulator thin films. *Electron. Struct.* **5**, 024002 (2023).

32. B. Fan, W. Duan, A. Rubio, P. Tang, Chiral Floquet engineering on topological fermions in chiral crystals. *NPJ Quantum Mater.* **9**, 101 (2024).

33. E. J. Sie, J. McIver, Y. H. Lee, L. Fu, J. Kong, N. Gedik, Valley-selective optical Stark effect in monolayer WS2. *Nat. Mater.* **14**, 290–294 (2015).

34. E. J. Sie, C. H. Lui, Y. H. Lee, L. Fu, J. Kong, N. Gedik, Large, valley-exclusive Bloch-Siegert shift in monolayer WS_2. *Science* **355**, 1066–1069 (2017).

35. J.-Y. Shan, M. Ye, H. Chu, S. Lee, J. G. Park, L. Balents, D. Hsieh, Giant modulation of optical nonlinearity by Floquet engineering. *Nature* **600**, 235–239 (2021).

36. X. Zhang, T. Carbin, A. B. Culver, K. Du, K. Wang, S.-W. Cheong, R. Roy, A. Kogar, Light-induced electronic polarization in antiferromagnetic Cr_2O_3. *Nat. Mater.* **23**, 790–795 (2024).

37. T. Oka, S. Kitamura, Floquet engineering of quantum materials. *Annu. Rev. Condens. Matter Phys.* **10**, 387–408 (2019).

38. S. Zhou, C. Bao, B. Fan, H. Zhou, Q. Gao, H. Zhong, T. Lin, H. Liu, P. Yu, P. Tang, S. Meng, W. Duan, S. Zhou, Pseudospin-selective Floquet band engineering in black phosphorus. *Nature* **614**, 75–80 (2023).

39. S. Zhou, C. Bao, B. Fan, F. Wang, H. Zhong, H. Zhang, P. Tang, W. Duan, S. Zhou, Floquet engineering of black phosphorus upon below-gap pumping. *Phys. Rev. Lett.* **131**, 116401 (2023).

40. C. Bao, F. Wang, H. Zhong, S. Zhou, T. Lin, H. Zhang, X. Cai, W. Duan, S. Zhou, Light-induced ultrafast glide-mirror symmetry breaking in black phosphorus. *ACS Nano* **18**, 32038–32044 (2024).

41. A. H. Castro Neto, F. Guinea, N. M. Peres, K. S. Novoselov, A. K. Geim, The electronic properties of graphene. *Rev. Mod. Phys.* **81**, 109–162 (2009).

42. Y. Chung, M. Kim, Y. Kim, S. Cha, J. W. Park, J. Park, Y. Yi, D. Song, J. H. Ryu, K. Lee, T. K. Kim, C. Cacho, J. Denlinger, C. Jozwiak, E. Rotenberg, A. Bostwick, K. S. Kim, Dark states of electrons in a quantum system with two pairs of sublattices. *Nat. Phys.* **20**, 1582–1588 (2024).

43. S. W. Jung, S. H. Ryu, W. J. Shin, Y. Sohn, M. Huh, R. J. Koch, C. Jozwiak, E. Rotenberg, A. Bostwick, K. S. Kim, Black phosphorus as a bipolar pseudospin semiconductor. *Nat. Mater.* **19**, 277–281 (2020).

44. C. Bao, M. Schüler, T. Xiao, F. Wang, H. Zhong, T. Lin, X. Cai, T. Sheng, X. Tang, H. Zhang, P. Yu, Z. Sun, W. Duan, S. Zhou, Manipulating the symmetry of photon-dressed electronic states. *Nat. Commun.* **15**, 10535 (2024).

45. O. Neufeld, D. Podolsky, O. Cohen, Floquet group theory and its application to selection rules in harmonic generation. *Nat. Commun.* **10**, 405 (2019).

46. M. E. Tzur, O. Neufeld, E. Bordo, A. Fleischer, O. Cohen, Selection rules in symmetry-broken systems by symmetries in synthetic dimensions. *Nat. Commun.* **13**, 1312 (2022).

47. G. Lerner, O. Neufeld, L. Hareli, G. Shoulga, E. Bordo, A. Fleischer, D. Podolsky, A. Bahabad, O. Cohen, Multiscale dynamical symmetries and selection rules in nonlinear optics. *Sci. Adv.* **9**, eade0953 (2023).

48. G. Wang, C. Li, P. Cappellaro, Observation of symmetry-protected selection rules in periodically driven quantum systems. *Phys. Rev. Lett.* **127**, 140604 (2021).

49. G. Engelhardt, J. Cao, Dynamical symmetries and symmetry-protected selection rules in periodically driven quantum systems. *Phys. Rev. Lett.* **126**, 090601 (2021).

50. L. Tao, A. Scrinzi, Photo-electron momentum spectra from minimal volumes: The timedependent surface flux method. *New J. Phys.* **14**, 013021 (2012).

51. U. De Giovannini, H. Hübener, A. Rubio, A first-principles time-dependent density functional theory framework for spin and time-resolved angular-resolved photoelectron spectroscopy in periodic systems. *J. Chem. Theory Comput.* **13**, 265–273 (2017).

52. U. De Giovannini, H. Hübener, S. A. Sato, A. Rubio, Direct measurement of electron-phonon coupling with time-resolved ARPES. *Phys. Rev. Lett.* **125**, 136401 (2020).

53. C. S. Kern, A. Haags, L. Egger, X. Yang, H. Kirschner, S. Wolff, T. Seyller, A. Gottwald, M. Richter, U. De Giovannini, A. Rubio, M. G. Ramsey, F. C. Bocquet, S. Soubatch, F. S. Tautz, P. Puschnig, S. Moser, Simple extension of the plane-wave final state in photoemission: Bringing understanding to the photon-energy dependence of two-dimensional materials. *Phys. Rev. Res.* **5**, 033075 (2023).

54. M. Ezawa, Topological origin of quasi-flat edge band in phosphorene. *New J. Phys.* **16**, 115004 (2014).

55. V. Tran, R. Soklaski, Y. Liang, L. Yang, Layer-controlled band gap and anisotropic excitons in few-layer black phosphorus. *Phys. Rev. B* **89**, 235319 (2014).

56. A. Rodin, A. Carvalho, A. Castro Neto, Strain-induced gap modification in black phosphorus. *Phys. Rev. Lett.* **112**, 176801 (2014).

57. J. Kim, S. S. Baik, S. W. Jung, Y. Sohn, S. H. Ryu, H. J. Choi, B.-J. Yang, K. S. Kim, Two-dimensional Dirac fermions protected by space-time inversion symmetry in black phosphorus. *Phys. Rev. Lett.* **119**, 226801 (2017).

58. A. Damascelli, Z. Hussain, Z.-X. Shen, Angle-resolved photoemission studies of the cuprate superconductors. *Rev. Mod. Phys.* **75**, 473 (2003).

59. X. Andrade, D. Strubbe, U. de Giovannini, A. H. Larsen, M. J. Oliveira, J. Alberdi-Rodriguez, A. Varas, I. Theophilou, N. Helbig, M. J. Verstraete, L. Stella, F. Nogueira, A. Aspuru-Guzik, A. Castro, M. A. Marques, A. Rubio, Real-space grids and the Octopus code as tools for the development of new simulation approaches for electronic systems. *Phys. Chem. Chem. Phys.* **17**, 31371–31396 (2015), 10.1039/c5cp00351b.

60. N. Tancogne-Dejean, M. J. T. Oliveira, X. Andrade, H. Appel, C. H. Borca, G. L. Breton, F. Buchholz, A. Castro, S. Corni, A. A. Correa, U. De Giovannini, A. Delgado, F. G. Eich, J. Flick, G. Gil, A. Gomez, N. Helbig, H. Hübener, R. Jestädt, J. Jornet-Somoza, A. H. Larsen, I. V. Lebedeva, M. Lüders, M. A. L. Marques, S. T. Ohlmann, S. Pipolo, M. Rampp, C. A. Rozzi, D. A. Strubbe, S. A. Sato, C. Schäfer, I. Theophilou, A. Welden, A. Rubio, Octopus, a computational framework for exploring light-driven phenomena and quantum dynamics in extended and finite systems. *J. Chem. Phys.* **152**, 124119 (2020).

61. C. Hartwigsen, S. Goedecker, J. Hutter, Relativistic separable dual-space Gaussian pseudopotentials from H to Rn. *Phys. Rev. B* **58**, 3641 (1998).

62. P. Li, I. Appelbaum, Electrons and holes in phosphorene. *Phys. Rev. B* **90**, 115439 (2014).

63. M. S. Dresselhaus, G. Dresselhaus, A. Jorio, *Group Theory: Application to the Physics of Condensed Matter* (Springer Science & Business Media, 2007).

64. U. D. Giovannini, H. Hübener, Floquet analysis of excitations in materials. *J. Phys. Mater.* **3**, 012001 (2020).
